# Psychometric properties of the sit-to-stand test for patients with pulmonary hypertension: A systematic review protocol

**DOI:** 10.1371/journal.pone.0275646

**Published:** 2022-10-05

**Authors:** Natália Lopes Cardoso, Joceline Ferezini de Sá, Larissa F. E. do Nascimento, Luciana A. Mendes, Selma Bruno, Rodrigo Torres-Castro, Guilherme A. F. Fregonezi, Vanessa R. Resqueti

**Affiliations:** 1 Laboratory of Technological Innovation in Rehabilitation and PneumoCardioVascular Lab/HUOL, Onofre Lopes University Hospital, Brazilian Company of Hospital Services (EBSERH), Federal University of Rio Grande do Norte (UFRN), Natal, Rio Grande do Norte, Brazil; 2 Laboratory of Cardiorespiratory and Metabolic Assessment—CORE/HUOL, Onofre Lopes University Hospital, Brazilian Company of Hospital Services (EBSERH), Federal University of Rio Grande do Norte (UFRN), Natal, Rio Grande do Norte, Brazil; 3 Department of Biomedical Engineering, Federal University of Rio Grande do Norte (UFRN), Natal, Rio Grande do Norte, Brazil; 4 Department of Physical Therapy, Faculty of Medicine, University of Chile, Santiago, Chile; Mugla Sitki Kocman Universitesi, TURKEY

## Abstract

**Background:**

Pulmonary hypertension (PH) is a complex syndrome characterized by increased pulmonary arterial pressure and classified into five groups, according to dyspnea on exertion and systemic muscle dysfunction. These symptoms can be identified using the sit-to-stand test (STS), which indirectly evaluates exercise tolerance and lower limb muscle strength. Previous studies used the STS in PH; however, psychometric properties to understand and validate this test were not described for patients with PH.

**Objective:**

To evaluate the psychometric properties (validity, reliability, and responsiveness) of different STS protocols in patients with PH.

**Methods and analyses:**

This is a systematic review protocol that will include studies using STS in patients with PH. Searches will be conducted on PubMed/MEDLINE, EMBASE, SciELO, Cochrane Central Register of Controlled Trials (CENTRAL), and Web of Science databases following PICOT mnemonic strategy and the Preferred Reporting Items for Systematic Reviews and Meta-Analyses Protocols (PRISMA-P). Rayyan software will be used for study selection. The Risk of bias will be assessed using the Consensus‐Based Standards for the Selection of Health Measurement Instruments (COSMIN) tool, while the quality of evidence will be assessed using the modified Grading of Recommendations, Assessment, Development, and Evaluation (GRADE). Two researchers will independently conduct the study, and a third researcher will be consulted in case of disagreement. The psychometric properties will be evaluated according to the COSMIN. This protocol was registered in the International Prospective Register of Systematic Reviews (PROSPERO, no. CRD42021244271).

**Conclusion:**

This systematic review will attempt to identify and show the available evidence on STS for different groups of PH and report validity, reliability, and responsiveness of different protocols.

## Introduction

Pulmonary hypertension (PH) is a complex clinical syndrome composed of progressive heterogeneous conditions that increase pulmonary arterial pressure and pulmonary vascular resistance. These symptoms are common to certain diseases that tend to evolve with right heart failure, reduced exercise tolerance, and early death of patient [1]. The current classification of PH is divided into five groups considering clinical data, pathophysiology, anatomopathological findings, and hemodynamic parameters [1–3].

Symptoms in patients with PH are non-specific and may lead to late diagnosis [4]. Progressive dyspnea on exertion is the earliest and most common symptom, reflecting the inability of the cardiovascular system to increase cardiac output during exertion. Other possible symptoms are fatigue, pre-syncope, chest pain, and palpitations [5]. At muscular level, patients present systemic muscle dysfunction and increased risk of functional decline due to loss of muscle function [6]. Exercise tolerance can be evaluated using different instruments, such as sit-to-stand test (STS) protocols [7, 8]. STS has several protocols, such as the five-repetition STS (5R-STS), the thirty-second STS (30S-STS), and the one-minute STS (1M-STS), and is also widely used to indirectly evaluate lower limb muscle strength [9], mobility, and functional independence [10].

STS evaluates exercise tolerance in several populations, including elderlies [11, 12] and patients with chronic respiratory conditions, such as chronic obstructive pulmonary disease [8, 13, 14], cystic fibrosis [15], and PH [16, 17]. Furthermore, reference values for healthy individuals [18] are widely used. Recently strong correlations were observed between 1M-STS and both daily step count and distance walked on the six-minute walk test (6MWT) in patients with PH [17]. Accordingly, this test is interesting and valuable, mainly because 1M-STS is easily performed, requires no special infrastructure, and induces less hemodynamic stress than 6MWT [19]. Thus, how the 30S-STS which a study also evaluated as reliable and valid for patients with PH [16]. Despite this easy application and clinical importance, there is no previous systematic review on the STS in the HP population, showing the gap found in the literature. This variety of STS protocols makes it important to understand the psychometric properties of the instruments used to measure exercise tolerance in HP. The evaluation of psychometric properties makes it possible to select and understand the instruments that will provide us with valid and reliable measurements [20], based on the Consensus-Based Standards for the Selection of Health Measurement Instruments which describes how properties should be handled and described [21].

Thus, this systematic review aims to identify the available evidence regarding STS in different PH groups and present psychometric properties (validity [criterion validity, construct validity], reliability [internal consistency and measurement error], and responsiveness) and number of repetitions achieved in different STS protocols. Results will help rehabilitation professionals safely interpret these measurements, supporting their use in clinical practice.

## Methods

### Registration and reporting of review findings

The systematic review will follow the Preferred Reporting Items for Systematic Reviews and Meta-Analyses Protocols (PRISMA-P) [22] (S1 Appendix). This protocol will also follow PICOT mnemonic strategy, while the risk of bias will be assessed using the Consensus-Based Standards for the Selection of Health Measurement Instruments (COSMIN) [23, 24]. The protocol was submitted and registered in the International Prospective Register of Systematic Reviews (PROSPERO, no. CRD42021244271).

### Eligibility criteria

Will be included studies of any type of randomized clinical trials or randomized controlled trials, besides observational studies (prospective, retrospective, longitudinal, or case-control) published in English. We will consider studies conducted at a hospital, outpatient, or primary care setting.

Exclusion criteria will be as follows: systematic reviews, *in vitro* studies, conference abstracts, theses, dissertations, literature reviews, studies conducted with children or mixed populations, and studies in which STS protocol did not meet the criteria described by Kahraman et al. (2020) [16], and those in which data from patients with PH were not analyzed separately or could not be extracted or obtained even after contacting authors.

### Characteristics of participants

We will include studies with adults participants with a clinical diagnosis of PH (i.e., resting mean pulmonary arterial pressure ≥ 25 mmHg or systolic pulmonary arterial pressure > 40 mmHg assessed using right cardiac catheterization or echocardiography) [3] or diagnosed by a specialist physician. Analyses will consider subgroups according to PH classification: pulmonary arterial hypertension, PH due to left heart disease, PH due to lung diseases or hypoxia or both, chronic thromboembolic PH and other pulmonary artery obstructions, and PH due to unknown or multifactorial mechanisms [1].

### Type of intervention

We will include studies that assess exercise tolerance using STS (30S-STS, 1M-STS, or 5R-STS), according to Kahraman et al. (2020) [16].

### Type of outcome measures

#### Primary outcomes

Psychometric properties (validity [criterion validity, construct validity], reliability [internal consistency and measurement error], and responsiveness) and repetitions achieved in STS protocols by participants with PH.

#### Secondary outcomes

- Association between STS and patient-reported outcomes (PROM) (e.g., symptoms, health-related quality of life, nocturnal symptoms, anxiety levels, and depression);
- Reported symptoms during STS protocols using Borg scale;
- Changes in respiratory or cardiovascular system in response to STS protocols before and after intervention (e.g., changes in peripheral arterial oxygen saturation or heart rate).

### Search strategy

Titles and abstracts will be screened on PubMed/MEDLINE, EMBASE, SciELO, Cochrane Central Register of Controlled Trials (CENTRAL), and Web of Science databases with no time restriction set for publications. We will use a combination of descriptors and the Medical Subject Headings (MeSH) to incorporate primary elements from the research question, including the construction, the population, and the type of intervention [21]. The searching strategy for all databases was added as a supplementary file with this protocol (S2 Appendix).

### Data collection and analysis

Two researchers (NLC and LN) will independently screen the titles and abstracts of the studies and will remove the irrelevant studies. A third researcher (JFS) will evaluate any discrepancies and will advise in case of disagreement. Studies screened will be inserted into Rayyan [25] software (pre-selection of duplicates) and confirmed manually according to eligibility criteria. Full text of eligible studies will be attached to Rayyan software and screened by the same researchers (NLC and LN). In case of disagreement, the third researcher (JFS) will be consulted. Reasons for exclusion will be recorded, and screening will be summarized in a PRISMA flowchart (S1 Fig).

### Data extraction and management

The COSMIN data extraction form related to measurement properties [21] will be used by two researchers (NLC and LN) to independently extract all data. We will solve any disagreements by discussion with a third researcher (JFS). Therefore, essential information will be extracted from studies to fill boxes from the checklist. The results will be quantitatively pooled or qualitatively summarized [21].

### Risk of bias assessment

Two researchers (NLC and LN) will independently analyze the methodological quality of studies using COSMIN risk of bias checklist [26, 27]. We will solve any disagreements on the risk of bias assessment by discussion with a third researcher (JFS). Each consistent result will be grouped quantitatively or summarized qualitatively and compared to criteria for good measurement properties, interpreted as very good, adequate, doubtful, or inadequate. Methodological quality of each single study on a measurement property will be included in a summary of findings table [27, 28]. Next, results of every single study on a measurement property will be rated against updated criteria for good measurement properties as sufficient (+), insufficient (-), or indeterminate (?) [26].

### Assessment of quality of evidence

The Grading of Recommendations, Assessment, Development, and Evaluation (GRADE) will be used to assess the quality of evidence of the systematic review. Evidence will be classified as high, moderate, low, or very low [26], based on risk of bias, inconsistency, imprecision, and indirectness [29].

### Data synthesis

We will use the Odds Ratio, Relative Risk or Risk Difference for the analysis of dichotomous data, for the continuous variables they will be presented as means, medians, and standard deviations. In the case of performing a quantitative synthesis (meta-analysis), we will use RevMan V.5.3.528 software for homogeneous studies. In case of missing data, we will contact the authors to obtain the data.

We plan to carry out subgroup analyses, with the type of PH and another subgroup analysis for the different STS protocols. If necessary, we will perform sensitivity analyses to examine the effects of methodological quality in the pooled estimate, removing studies that are rated at high risk of bias.

## Discussion

Patients with PH present exercise intolerance; therefore, STS is a simple and advantageous instrument because protocols are faster and elicit less hemodynamic stress than other field tests. The systematic review will identify validity (criterion validity, construct validity), reliability (internal consistency and measurement error), and responsiveness.

These properties will also help interpret and compare STS and field tests (e.g., 6MWT) in clinical practice of these patients. In addition, identifying STS as a valid, reproductive, and responsive measure may also increase knowledge regarding intrinsic characteristics of the test in different types of PH and support better indication of STS characteristics and adaptation of patients with PH to exercise. Thus, this systematic review will determine the importance of STS to develop standard protocols and improvements health services.

## Supporting information

S1 Fig Flow diagram—PRISMA 2020. (TIF)

S1 Appendix PRISMA-P 2015 checklist. (DOCX)

S2 Appendix Search strategy. (DOCX)
