# Supplementary figures and images for: Psychometric properties of the sit-to-stand test for patients with pulmonary hypertension: A systematic review protocol
Source: PLoS One. 2022 Oct 5;17(10):e0275646. doi: 10.1371/journal.pone.0275646 (PMC9534407; doi:10.1371/journal.pone.0275646)

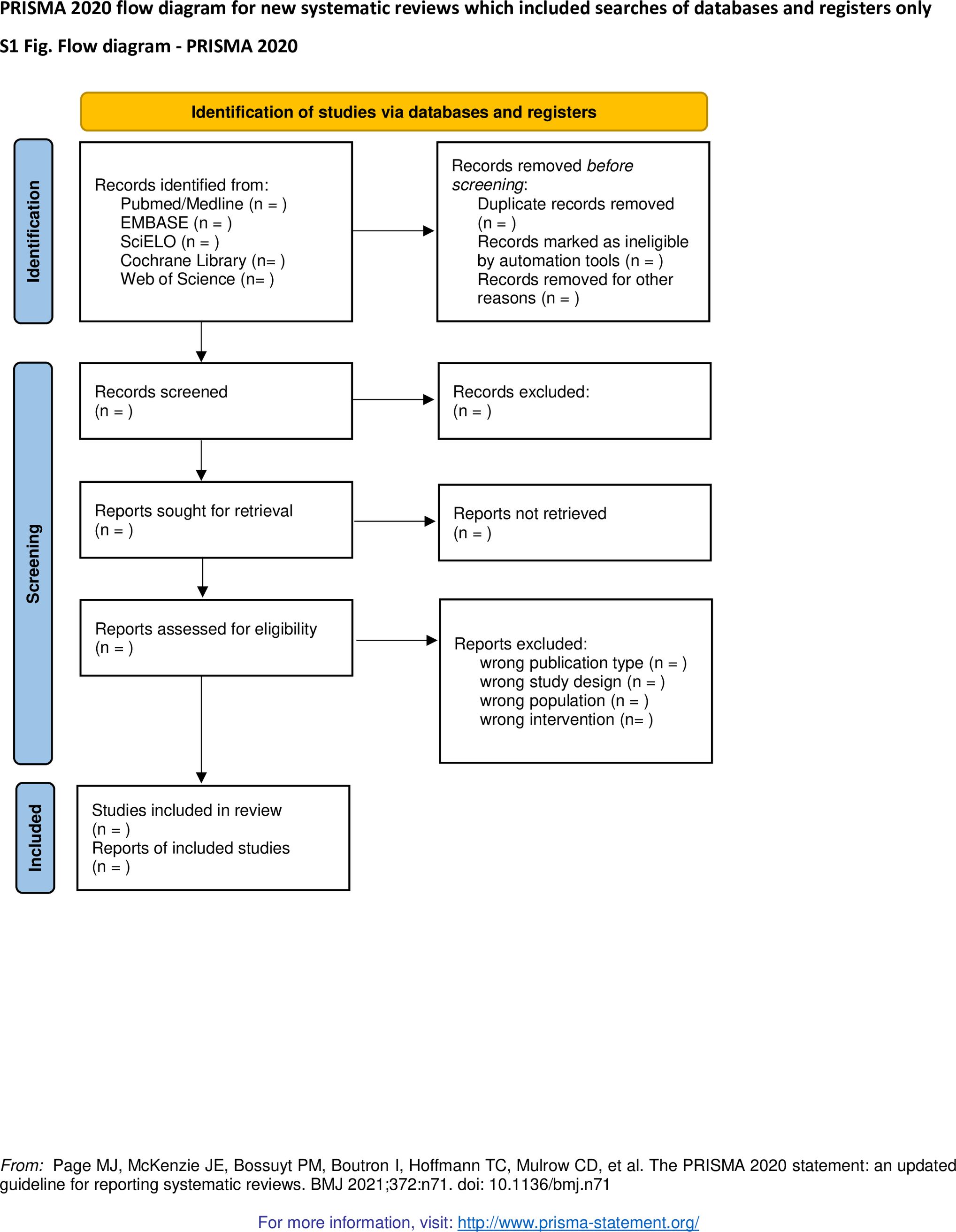

Supplement: S1 Fig — (TIF) [file pone.0275646.s001.tif]
