# Supplementary material for: Psychometric properties of the sit-to-stand test for patients with pulmonary hypertension: A systematic review protocol
Source: PLoS One. 2022 Oct 5;17(10):e0275646. doi: 10.1371/journal.pone.0275646 (PMC9534407; doi:10.1371/journal.pone.0275646)
Supplement: S2 Appendix — (DOCX) [file pone.0275646.s003.docx]

**S2 Appendix - Search strategy**

**Pubmed**

Pulmonary Hypertension OR Heritable Pulmonary Arterial Hypertension OR Pulmonary Hypertension, Primary, 1 OR Idiopathic Pulmonary Hypertension OR Pulmonary Heart Diseases OR Right Ventricular Systolic Pressure OR Pulmonary Vascular Disease OR Pulmonary Artery Systolic Pressure OR pulmonary arterial hypertension OR Chronic thromboembolic pulmonary hypertension

AND

Sit-to-stand test OR five-repetition sit-to-stand test OR 30 seconds sit-to-stand test OR 1-minute sit-to-stand test OR Exercise tolerance OR Exercise test OR physical activity OR Short Physical Performance Battery test

**EMBASE**

"Pulmonary Hypertension" OR "Heritable Pulmonary Arterial Hypertension" OR "Pulmonary Hypertension, Primary, 1" OR "Idiopathic Pulmonary Hypertension" OR "Pulmonary Heart Diseases" OR "Right Ventricular Systolic Pressure" OR "Pulmonary Vascular Disease" OR "Pulmonary Artery Systolic Pressure" OR “Pulmonary Arterial Hypertension” OR “Chronic thromboembolic pulmonary hypertension”

AND

"Sit-to-stand test" OR "five-repetition sit-to-stand test" OR "30 seconds sit-to-stand test" OR "1-minute sit-to-stand test" OR "Exercise tolerance" OR "Exercise test" OR "physical activity" OR "Short Physical Performance Battery test"

**SciELO**

(*Hipertensão pulmonar) OR (Hipertensão arterial pulmonar) OR (Pressão arterial pulmonar) OR (Pressão sistólica da artéria pulmonar) OR (Doença cardíaca pulmonar) OR (Doença vascular pulmonar) OR (Pressão sistólica ventricular direita) OR (Hipertensão arterial pulmonar primária) OR (Hipertensão arterial pulmonar hereditária) OR (Hipertensão Arterial Pulmonar Idiopática) OR (Chronic thromboembolic pulmonary hypertension)

AND

(teste senta e levanta) OR (teste de senta e levanta de cinco repetições) OR (teste de senta e levanta de 30 segundos) OR (teste de senta e levanta de 1 minuto) OR (Exercício de tolerância) OR (Teste de esforço) OR (Atividade física) OR (teste de desempenho físico de curto bateria)

**Cochrane Library**

"Pulmonary Hypertension" OR "Heritable Pulmonary Arterial Hypertension" OR "Pulmonary Hypertension, Primary, 1" OR "Idiopathic Pulmonary Hypertension" OR "Pulmonary Heart Diseases" OR "Right Ventricular Systolic Pressure" OR "Pulmonary Vascular Disease" OR "Pulmonary Artery Systolic Pressure" OR “pulmonary arterial hypertension” OR “Chronic thromboembolic pulmonary hypertension”

AND

"Sit-to-stand test" OR "five-repetition sit-to-stand test" OR "30 seconds sit-to-stand test" OR "1-minute sit-to-stand test" OR "Exercise tolerance" OR "Exercise test" OR "physical activity" OR "Short Physical Performance Battery test"

**Web Of Science**

TS = (Pulmonary Hypertension OR Heritable Pulmonary Arterial Hypertension OR Pulmonary Hypertension, Primary, 1 OR Idiopathic Pulmonary Hypertension OR Pulmonary Heart Diseases OR Right Ventricular Systolic Pressure OR Pulmonary Vascular Disease OR Pulmonary Artery Systolic Pressure OR pulmonary arterial hypertension OR Chronic thromboembolic pulmonary hypertension)

AND

TS = (Sit-to-stand test OR five-repetition sit-to-stand test OR 30 seconds sit-to-stand test OR 1-minute sit-to-stand test OR Exercise tolerance OR Exercise test OR physical activity OR Short Physical Performance Battery test)
